# Breast density assessment via quantitative sound-speed measurement using conventional ultrasound transducers

**DOI:** 10.1007/s00330-024-11335-w

**Published:** 2025-01-11

**Authors:** Can Deniz Bezek, Monika Farkas, Dieter Schweizer, Rahel A. Kubik-Huch, Orcun Goksel

**Affiliations:** 1 Department of Information Technology, Uppsala University, 75237 Uppsala, Sweden; 2 Department of Radiology, Kantonsspital Baden, affiliated Hospital for Research and Teaching of the Faculty of Medicine of the University of Zurich, 5404 Baden, Switzerland; 3 Computer-Assisted Applications in Medicine, ETH Zurich, 8092 Zurich, Switzerland

**Keywords:** Ultrasonography, Breast density, Breast neoplasm, Mammography

## Abstract

**Objectives:**

The aim is to assess the feasibility and accuracy of a novel quantitative ultrasound (US) method based on global speed-of-sound (g-SoS) measurement using conventional US machines, for breast density assessment in comparison to mammographic ACR (m-ACR) categories.

**Materials and methods:**

In a prospective study, g-SoS was assessed in the upper-outer breast quadrant of 100 women, with 92 of them also having m-ACR assessed by two radiologists across the entire breast. For g-SoS, ultrasonic waves were transmitted from varying transducer locations and the image misalignments between these were then related analytically to breast SoS. To test reproducibility, two consecutive g-SoS acquisitions each were taken at two similar breast locations by the same operator.

**Results:**

Measurements were found highly repeatable, with a mean absolute difference ± standard deviation of 3.16 ± 3.79 m/s. Multiple measurements were combined yielding a single g-SoS estimate per each patient, which strongly correlated to m-ACR categories (Spearman’s = 0.773). The g-SoS values for categories A-D were 1459.6 ± 0.74, 1475.6 ± 15.92, 1515.6 ± 27.10, and 1545.7 ± 20.62, with all groups (except A-B) being significantly different from each other. Dense breasts (m-ACR C&D) were classified with 100% specificity at 78% sensitivity, with an area under the curve (AUC) of 0.931. Extremely dense breasts (m-ACR D) were classified with 100% sensitivity at 77.5% specificity (AUC = 0.906).

**Conclusion:**

Quantitative g-SoS measurement of the breast was shown feasible and repeatable using conventional US machines, with values correlating strongly with m-ACR assessments.

**Key Points:**

***Question*** *Breast density is a strong predictor of risk for breast cancer, which frequently develops in dense tissue regions. Therefore, density assessment calls for refined non-ionizing methods.*

***Findings*** *Quantitative global speed-of-sound (g-SoS) measurement of the breast is shown to be feasible using conventional US machines, repeatable, and able to classify breast density with high accuracy.*

***Clinical relevance*** *Being effective in classifying dense breasts, where mammography has reduced sensitivity, g-SoS can help stratify patients for alternative modalities. Ideal day for mammography or MRI can be determined by monitoring g-SoS. Furthermore, g-SoS can be integrated into personalized risk assessment.*

**Graphical Abstract:**

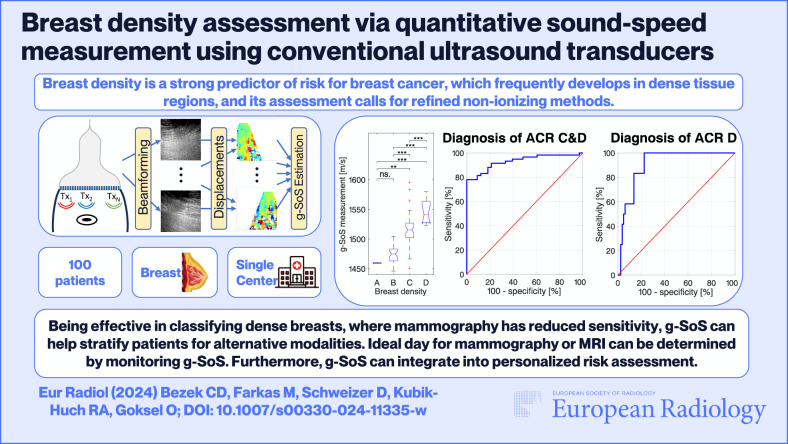

## Introduction

Breast density refers to the amount of glandular tissue observed on mammography images. The reporting is typically done following the guidelines of the American College of Radiology (ACR) Breast Imaging Reporting and Data System (BI-RADS)—the latest being the 5th edition [1]. This system categorizes breast density into four classes: A (almost entirely fatty), B (scattered areas of fibroglandular density), C (heterogeneously dense, potentially obstructing small masses), and D (extremely dense, associated with reduced mammography sensitivity) [1]. One of the leading causes of cancer-related mortality in women is breast cancer [2]. Breast density is a strong predictor of risk for breast cancer [3, 4], as women with very dense breasts were reported to have a comparatively much higher risk of developing cancer [4–6]. Therefore, breast density has emerged as an important factor in personalized breast cancer risk assessment, in addition to other factors such as family history and hormone therapy [7–9]. Several recent regulatory changes thus underscore the importance of breast density and reflect its acknowledgment as an independent risk factor for breast cancer: In the United States, as of December 2019, 38 states have enacted laws requiring health professionals to inform women with increased breast density (or, in some states, all women) about their breast density [10]. In October 2023, the Food and Drug Administration introduced regulations (took effect in September 2024) requiring mammography reports that are given to patients to indicate if the patient has dense breasts while also explaining the significance of breast density [11].

The association between dense breast tissue and an elevated risk of breast cancer calls for refined screening methods [6, 12, 13]. Several studies [6] reported the odds of developing breast cancer being 2.9 to 6.0 times higher for the densest breasts compared to the least dense breasts. Despite variation in density category definitions, a 4.0 or greater increase in the odds of developing cancer for very dense breasts has often been reported in the literature [5]. Furthermore, with increasing breast density, the sensitivity of mammography is reduced [14, 15], from 87% for low breast density down to 63% for high breast density [16]. Therefore, patients with high mammographic breast density may be required to undergo additional examinations, such as ultrasound (US) or breast magnetic resonance imaging (MRI), to improve diagnostic sensitivity [17]. Such limitations of mammography have further prompted investigations into new imaging technologies for breast cancer assessment, for which the US offers non-ionizing and cost-effective alternatives [18–24]. The latest BI-RADS lexicon [1] categorizes B-mode US appearance of the breast tissue into: a (homogeneous background echotexture-fat), b (homogeneous background echotexture-fibroglandular), and c (heterogeneous background echotexture). Such assessment, however, is entirely qualitative and can therefore be highly subjective.

US computed tomography has shown promise in measuring breast density and detecting cancer through speed-of-sound (SoS) measurements [18–22]. However, a drawback of this technology is its need for a dedicated US system and a custom patient table with a water bath in which the breast is submersed during scanning. It then requires additional preparation time, logistic planning, space, and a specialized operator, all of which are scarce resources and hence hinder application in the clinics. A hand-held transducer with an acoustic reflector add-on was used for breast density characterization in [23], but this method requires hardware attached to the probe and hence reduces the flexibility in probe placement and manipulation. A conventional transducer without any additional equipment was used in pulse-echo mode for the differential diagnosis of breast cancer [24]. Although the study was conducted with a small (20) group of patients, it showed promising results in differentiating ductal carcinoma from fibroadenoma and healthy breast tissue.

Quantitative US biomarkers are valuable to incorporate in standard US systems with traditional hand-held array transducers working in pulse-echo mode. For the breast, such imaging biomarkers can serve as complementary or alternative to mammography. This is particularly important for breast density assessment considering the conventional reliance on the radiographer’s interpretation from mammography. An analytical method for global SoS (g-SoS) estimation using standard hand-held transducers was proposed in [25] and evaluated in numerical and tissue-mimicking phantoms. The goal of this study was to assess the feasibility, robustness, and accuracy of a novel quantitative US method based on g-SoS measurement using conventional US machines, for breast density assessment in comparison to mammographic ACR (m-ACR) categories.

## Materials and methods

### Study design

**Fig. 1:**
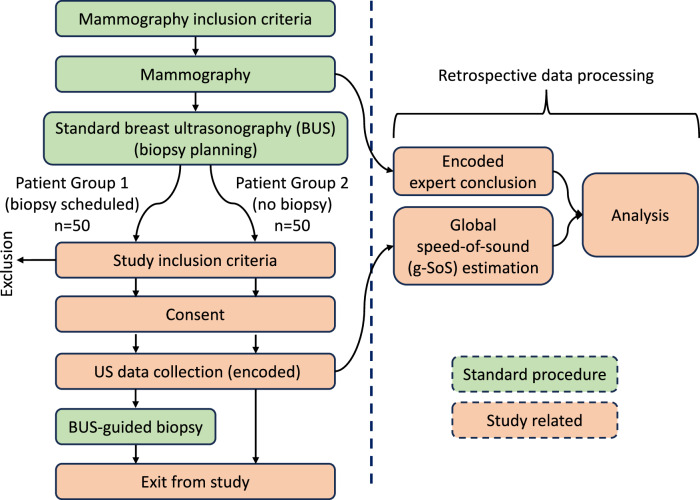
Flowchart of the study

The current study, NCT04480437, is a prospective, single-institution study in which 100 female patients who were scheduled for diagnostic breast examination between January and December 2021 at Kantonsspital Baden (Switzerland), who met the inclusion criteria and whom we approached for admission were included. To encourage variety in the dataset and avoid bias toward breast density, during admission, we aimed to balance the patient cohort half-half (equally) between those with foreseen biopsies and those without. The study received approval from the local ethics committee (EKNZ) and the Swiss authority for medical devices (Swissmedic). Informed written patient consent was obtained from all women prior to the examination. Patients included in this study were women aged 18 or older, and with sufficient German language skills to comprehend written and verbal information about the study-related process. Lactating or vulnerable patients, and patients with mastitis were exclusion criteria. No selection was made based on the patient’s breast density. The mean age of the patients was 53.8, ranging from 23 to 85 years. A study flowchart is given in Fig. 1.

**Fig. 2:**
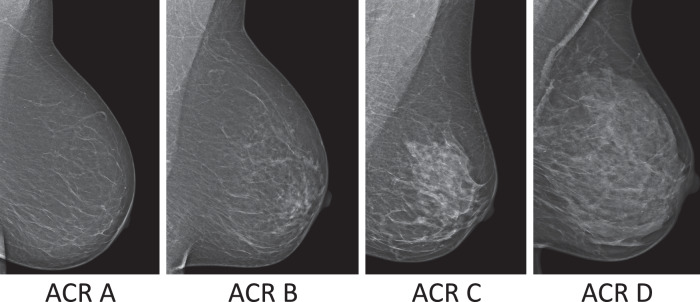
Sample mammography images for density categories based on American College of Radiology (ACR) categorization, from the least dense (ACR A) to the most dense (ACR D). All images are obtained using Siemens Healthineers MAMMOMAT Revelation

All 100 patients underwent a US examination, performed by the same two radiologists. From these, a total of 92 patients underwent mammography imaging (Siemens Healthineers, MAMMOMAT Revelation) for diagnostic purposes. Eight patients were excluded from mammography, either due to young age (4 were under the age of 27) or based on previous recommendations or US findings. Sample images for different m-ACR categories are shown in Fig. 2. Two radiologists performed independent breast density assessments in mammography. The first reader was the radiologist who performed the US examination, and the second reader later assessed the mammography images independently. If these assessments did not agree, a third radiology specialist assessed the image as a tiebreaker. This happened for 3 out of the 92 patients in this study when the first two readers disagreed between ACR A and B or ACR C and D.

Menopausal status of all patients was scrutinized (pre- or postmenopausal). Unknown hormonal status was reported for 16 patients, due to hysterectomy, menstruation at irregular intervals, contraceptive pills, or intrauterine device (IUD). Thirteen patients were receiving hormonal replacement. Clinical characteristics and patient profiles are summarized in Table 1.

**Table 1:** Clinical characteristics and patient profile of the study participants

ACR breast density class	A	B	C	D	No mammography	Overall
Number of patients (#)	2	31	47	12	8	100
Mean age (years)	70.5	61.1	54.3	45.2	31.1	53.8
Premenopausal (#)	0	3	16	6	5	30
Postmenopausal (#)	2	24	25	1	0	52
Unknown menopausal (#)	0	4	6	5	3	18
Hormone replacement (#)	0	1	8	3	2	14

### Pulse-echo global speed-of-sound estimation technique

**Fig. 3:**
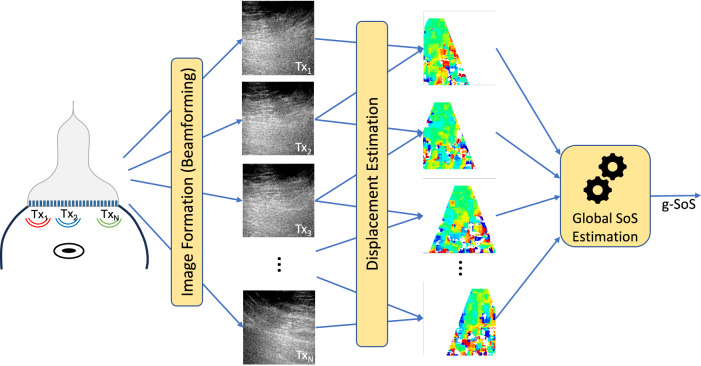
Overview of the global speed-of-sound (g-SoS) estimation technique

A global SoS value for the tissue is quantified using the method of [25], as illustrated in Fig. 3. The tissue is first insonified multiple times using US waves that are emitted from different locations on the transducer surface. From the received US echoes, separate images are then generated (beamformed) for each transmission. For converting the echo arrival times to distances and locations on the B-mode image, this beamforming step requires the assumption of an SoS value, for which typically an average and generic tissue SoS value is used in US B-mode imaging. Similarly, we start here with such a generic SoS value to beamform images first, and then compare these images for any minute disparities and misalignments. If the assumed and the actual SoS values are the same, looking at a tissue point from different directions should not make a major difference. Note that in this setting, the waves originating from two different emissions and arriving at any observed tissue location travel through different paths and distances, e.g., an image point on the right is closer to a transmission origin to the right. If the actual tissue SoS and the assumed beamforming SoS differ, the geometric distance differences then cause a systematic shift (misalignment) between the beamformed US images. These minute misalignments, which are often imperceptible to the eye, are here tracked using fine-level displacement tracking techniques. Then, using a geometric model for the path differences, the actual SoS is predicted. For a robust estimation, we perform this analytical estimation for multiple transmission image pairs concurrently. To ascertain the estimated SoS value, we repeat the above process by replacing the assumed SoS value with the estimated, iterating until the SoS values between successive iterations change less than a SoS precision threshold of 1 m/s or up to a maximum of 10 iterations, whichever comes first.

### Pulse-echo data collection during breast examination

Each breast US was performed by the first reader using a customized UF-760AG US system by (Fukuda Denshi). A linear array transducer (FUT-LA385-12P) with 128 elements and 300 µm pitch was used. Each transmit event contained 4 half-cycle pulses with a center frequency of 5 MHz. An imaging sequence based on virtual-source diverging waves is used for data acquisition, given their higher signal-to-noise ratio and robustness to motion [26]. For each data acquisition, 17 US transmit events were emitted, corresponding images beamformed up to a depth of 3 cm, and displacements were calculated as in [26]. Using these, g-SoS is estimated using the method of [25], also summarized above. The beamforming time offset was calibrated using tissue-mimicking phantoms with known SoS values.

The US-imaged breast location was the upper-outer quadrant (UOQ), where two images were taken at the same location, these being perpendicular to each other. During the data collection, the radiologist first used a standard US machine (GE Healthcare, Logic E10) present on-site to conduct standard US breast examination. Afterwards, on the breast side (left or right) with any detected lesion, the radiologist used the GE US system to acquire B-mode images for the purpose of guiding the probe to a location characterized by homogeneous tissue distribution. Once the location was determined based on the GE system, the UF-760AG transducer was placed horizontally at that location for a transversal view of the UOQ, and the data collection for our method was initiated using a foot pedal while keeping the transducer as steady as possible. We implemented such pedal-activated collection to acquire two consecutive *acquisitions* within a fraction of a second. Next, the transducer was rotated in a rolling motion, imaging the same UOQ region but this time in a vertical orientation for a sagittal view; collecting another two acquisitions. For 4 patients with lesions in both breasts, g-SoS assessments were arbitrarily taken from the left side for comparison with m-ACR. We collected our data using the Fukuda machine, as this allows us to access unprocessed (raw) US channel signal data, which is required by our method and currently not available on the GE system. To avoid changing or affecting the existing clinical routine, we chose not to replace the GE machine entirely but instead to include the Fukuda system as an additional imaging system. The collected US data was processed retrospectively to compute g-SoS measurements; hence no results were known to the readers throughout their US or mammography assessments. Accordingly, in total, four g-SoS measurements of the same breast region of each patient were available for further analysis, with two measurement pairs each being from the exact same location.

The discrepancy of the two measurements from the same location is first used to evaluate and report repeatability. Additionally, such discrepancy was utilized to judge which of the two pairs is more reliable in inferring the patient g-SoS as follows: Of the two pairs, the one with a larger measurement discrepancy is discarded, and the patient g-SoS is calculated as the average g-SoS of the remaining, more-consistent measurement pair.

### Statistical analysis

Statistical analysis was performed using MATLAB (2021b, The MathWorks Inc.). SoS variations between different acquisitions were assessed using intraclass correlation coefficient (ICC) and Bland-Altman [27] plots. In [28], ICC is defined as *poor* for ICC $<$ 0.5, *moderate* for 0.$5$ ≤ ICC < 0.75, *good* for 0.$75$ ≤ ICC < 0.9, and *excellent* for ICC ≥ 0.9. Bland-Altman plots were employed to illustrate the mean SoS difference and limits of agreement, i.e., mean SoS difference ± 1.96 standard deviations. SoS differences among various m-ACR categories were assessed through one-way analysis of variance (ANOVA), followed by the Tukey’s honestly significant difference (HsD) test. Statistical significance was reported with “***” for *p*-value ≤ 0.001, with “**” for 0.001 < *p*-value ≤ 0.01, with “*” for 0.01 < *p*-value < 0.05, and with “ns” for no statistical significance for higher *p*-values. The correlation between SoS and breast density categories was evaluated using the nonparametric Spearman’s rank correlation coefficient *r*. According to [29], correlation is *negligible* for *r* < 0.2, *weak* for 0.2 ≤ *r* < 0.4, *moderate* for 0.4 ≤ *r* $<$ 0.7, *strong* for 0.7 ≤ *r* $<$ 0.9, and *very strong* for *r* ≥ 0.9. Receiver operating characteristic (ROC) analysis was conducted to assess the diagnostic accuracy of SoS for different m-ACR-determined breast densities. The evaluation included reporting sensitivity, specificity, and the area under the curve (AUC). A linear regression model was fitted to examine the correlation between age and SoS, with the strength of this relationship quantified via *R*-square (*R*^2^).

## Results

We had g-SoS measurements from all 100 patients, whereas m-ACR categories only from the subset of 92 who had mammography exams. Accordingly, we used this subset for analyses that require breast density gold-standard, and the entire set otherwise.

### Repeatability of global SoS estimation in the breast

**Fig. 4:**
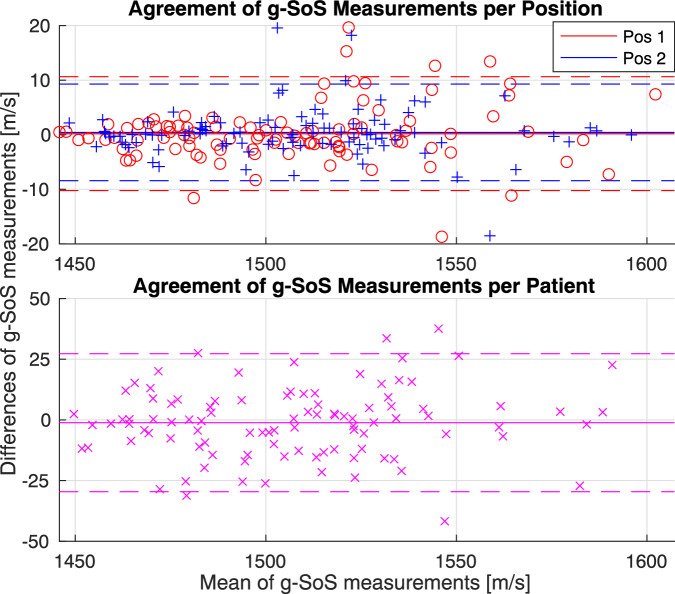
Agreement of global speed-of-sound (g-SoS) measurements per position and per patient. Solid lines show the mean of the measurement difference. Dashed lines show the limits of agreement, i.e., mean ± 1.96 * standard deviation

Measurement repeatability was assessed within the pairs, i.e., with the difference (discrepancy) between consecutive two acquisitions at the exact same location. With two measurement pairs per patient, one in each orientation, there are a total of 200 discrepancies reported in Fig. 4 (top) as color-coded per measurement orientation. For the first measurement position (orientation), the mean estimation difference and standard deviation were 0.20 m/s and 5.33 m/s, respectively. The mean absolute difference (MAD) between the two estimations was 3.53 m/s. In the second measurement position, the mean estimation difference ± standard deviation was 0.43 ± 4.52 m/s; with an MAD of 2.78 m/s. The ICC value was 0.99 for both measurement positions, 1 and 2.

By averaging the values from each acquisition pair, a per-patient per-position value is obtained. With these, the repeatability of g-SoS across different measurement positions/orientations of the same tissue region (upper-outer breast quadrant) was evaluated in Fig. 4 (bottom). Estimation difference means and standard deviation were –1.15 m/s and 14.50 m/s, with a MAD of 11.05 m/s and ICC of 0.91.

### Global SoS to predict mammographic ACR breast density class

#### g-SoS values for different breast density classes

**Fig. 5:**
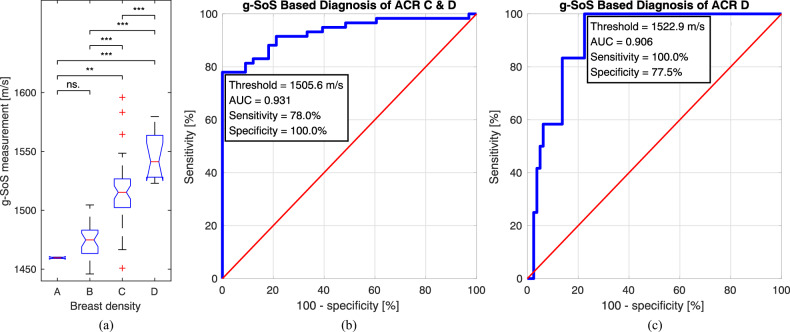
**a** Global speed-of-sound (g-SoS) measurements for different breast density classes. Statistical significance between different breast density groups is reported using a two-sample *t*-test with “***” for *p*-value ≤ 0.001, with “**” for 0.001 < *p*-value ≤ 0.01, and with “ns.” for no statistical significance with *p*-value > 0.01. **b** Receiver operating characteristic (ROC) analysis using g-SoS as a diagnostic biomarker for classifying dense (ACR C&D) breasts. **c** ROC analysis using g-SoS as a diagnostic biomarker for classifying very dense (ACR D) breasts

There was a strong correlation between m-ACR categories and quantified g-SoS values with *r* = 0.773. The mean g-SoS values for each m-ACR class are tabulated in Table 2. The distribution of g-SoS for each m-ACR group is shown in Fig. 5a. An ANOVA analysis demonstrated strongly significant differences across all four breast density categories. Pairwise comparisons between group means (*p*-values indicated with stars in Fig. 5) revealed strong evidence for differences, except for the A-B group difference (*p*-value = 0.77).

**Table 2:** Global speed-of-sound (g-SoS) measurements (mean ± standard deviation) for patients in each mammographic American College of Radiology (m-ACR) breast density category

m-ACR breast density	g-SoS (m/s)
m-ACR A (*N* = 2)	1459.6 ± 0.74
m-ACR B (*N* = 31)	1475.6 ± 15.92
m-ACR C (*N* = 47)	1515.6 ± 27.10
m-ACR D (*N* = 12)	1545.7 ± 20.62

#### g-SoS for breast density classification

ROC analyses were performed to assess g-SoS as a quantitative diagnostic biomarker for both classifying dense breasts (i.e., m-ACR C&D as positive vs. A&B as negative categories) and for classifying extremely dense breasts (i.e., D as positive vs. the other three as negative). For dense breast classification, AUC was 0.931, and for an SoS threshold of 1505.6 m/s, sensitivity was 78% at 100% specificity. For extremely dense breast classification, AUC was 0.906, and for 1522.9 m/s, threshold specificity was 77.5% at 100% sensitivity.

As an m-ACR predictor (classifier) from g-SoS measurements, we chose the above thresholds for B–C and C–D differentiation thresholds. For A-B, we choose 1460.2 m/s, which maximizes class A specificity. Based on this, the accordance between m-ACR and g-SoS-based breast density categories is shown in Table 3. All 31 patients with non-dense (A or B) breasts were accurately classified as either A or B with g-SoS. Moreover, all 12 patients with extremely dense (D) breasts were correctly classified as class D using g-SoS. Reanalyzing g-SoS-based diagnostic classification by permitting one category error (indicated by ± 1 category), 98.9% accuracy is reached with only one misclassification.

**Table 3:** Cross-tabulation of ultrasound global speed-of-sound (g-SoS) based prediction of mammographic American College of Radiology (m-ACR) breast density categories (as percentage in parentheses)

g-SoS-based density category	A	B	C	D	A or B	C or D	± 1 margin category
m-ACR A (*N* = 2)	2 (100%)	0	0	0	2 (100%)	0	2 (100%)
m-ACR B (*N* = 31)	4 (12.9%)	27 (87.1%)	0	0	31 (100%)	0	31 (100%)
m-ACR C (*N* = 47)	1 (2.1%)	13 (27.7%)	15 (31.9%)	18 (38.3%)	14 (29.8%)	33 (70.2%)	46 (97.9%)
m-ACR D (*N* = 12)	0	0	0	12 (100%)	0	12 (100%)	12 (100%)

With a margin of ± 1 category, a g-SoS-based prediction of a category is considered correct also for breasts in its two neighboring m-ACR categories

### B-mode visual assessment for breast density classification

We also studied the relation of B-mode US appearance assessments to breast density. Visual assessments (a/b/c) of the 92 B-mode examinations by the performing radiologist do not correlate with m-ACR categories (Spearman’s *r* = 0.133).

### SoS correlation with age

**Fig. 6:**
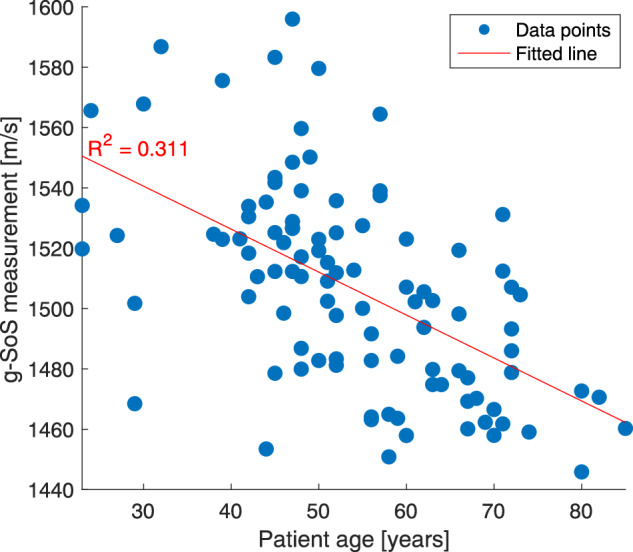
Global speed-of-sound (g-SoS) measurements with respect to age

Due to postmenopausal changes in glandular breast tissue, the breast density typically decreases with increasing age [30]. For all 100 patients enrolled, the distribution of SoS values with different ages are shown in Fig. 6. This indicates a moderate, negative, linear relationship (*r* = −0.581) between g-SoS and age.

## Discussion

In this study, we evaluate a quantitative US method (g-SoS) for assessing breast density based on global SoS measurement, which is found to be highly repeatable and strongly correlated with m-ACR categories. Dense breasts and extremely dense breasts were successfully classified with high accuracy. This study shows that quantitative global SoS measurement with standard US hardware and conventional hand-held transducers can effectively differentiate between different breast density categories.

Our results show that, while visual B-mode assessments do not correlate with m-ACR categories (*r* = 0.133), there is a significant correlation and accordance between m-ACR categories and g-SoS measurements (*r* = 0.773) similar to that reported in [23] (*r* = 0.746). This corroborates the correlations observed between volume-averaged SoS values from 3D US computed tomography and mammography percent density [20, 22]. In [31] the patients classified as density type 4 (dense breast) were found to have a significantly higher breast SoS value compared to those categorized as density type 1 (fatty), 2, and 3; classified according to the BI-RADS 4th edition that used numbers instead of letters. In contrast to these US-based approaches, the method utilized here only requires conventional US hardware and does not necessitate any additional physical components. Note that during US examination we used the FDA- and CE-certified UF-760AG for collecting raw data, which we later processed using the described methods to measure global SoS in the breast, which is then an off-label use of this device.

In this study, for extremely dense breast (m-ACR D) classification using g-SoS, the AUC was 0.906, reflecting a high level of diagnostic accuracy. With the chosen g-SoS threshold, all extremely dense breasts were identified correctly with 100% accuracy, as seen in Table 3. The AUC for dense (C or D) breast classification was 0.931, further indicating a high accuracy. With the chosen g-SoS threshold, all non-dense breasts (m-ACR A or B) were correctly classified as non-dense. This is a major improvement over [23], where 17% of patients with non-dense breasts (6/36) were incorrectly classified as dense breasts (C or D). Furthermore, at 100% specificity, the method presented here demonstrates a sensitivity of 78%, which is substantially higher compared to the 15% reported in [23].

Breast density is known to decrease with aging. Our results show a moderate, negative linear relationship (*r* = –0.581) between quantitative g-SoS and age. This corroborates the findings of [30], where an inverse relationship between age and mammographic breast density was shown with 7007 participants. In that study, a sizeable proportion of young women exhibited predominantly fatty breasts, while a subgroup of older women showed extremely dense breasts [30]. This aligns with our observations from Fig. 6, where some young patients present g-SoS values lower than some elderly patients. Hence, age is not a surrogate for breast density, further emphasizing the need for its independent assessment and reporting.

We found that SoS measurements were highly repeatable at both assessed positions, with an ICC of 0.988 for position 1 and 0.991 for position 2; comparable to those intrareader agreements reported in [23] (ICC = 0.966–0.994). When comparing measurements taken at different orientations of the same breast anatomy, the g-SoS repeatability was also good, with an ICC of 0.908. Notably, these ICC values are considerably superior to intrareader agreements reported for ACR mammography breast density, e.g., ICC = 0.77 in [32] or inter-reader agreements (ICC = 0.57 [32] and ICC = 0.731–0.774 [23]). Being an automatic and quantitative method, our presented approach is inherently more robust to reader variability. Note that the goal of our repeatability study was not to investigate the repeatability of readings throughout a breast, but rather to test the robustness and repeatability of multiple measurements and different probe orientations around the same location, the UOQ in this case. Our results indicate that the g-SoS-based density measurement from the UOQ alone gives comparable results to X-ray mammography-based readings. This finding highlights the clinical applicability of g-SoS at a single indicative location (UOQ).

The clinical use of the terminology *breast density* refers mainly to the amount of glandular tissue observed. Breast epithelium and stroma attenuate X-rays more than fat and hence appear whiter on mammograms compared to fat [6]. From a medical physics and biomechanics perspective, SoS in a medium is a function of the medium’s (bulk) stiffness and density, where SoS is proportional to stiffness but *inversely* proportional to density. According to this, for higher breast (physical) density, a *lower* SoS should be observed. This then appears to contradict the findings herein as well as several earlier works. Nevertheless, this can be explained considering that the glandular tissues are expected to also have higher stiffness compared to fat. The empirical observation that SoS is higher in clinically denser breasts indicates that the breast (bulk) stiffness is increasing more than its (biomechanical) density. Differentiating these two biomechanical variables and potentially assessing their comparative value as a cancer risk factor independently can be an interesting future research direction. Such differentiation nevertheless requires other concurrent measurement modalities (e.g., Hounsfield units to factor out physical density) and/or ex-vivo biomechanical experiments on excised breast tissues, as in [33, 34], which are beyond the focus of our current work.

Our study has a few limitations. Our cohort includes a few women younger than 40 and a few on hormone replacement therapy, so efficacy in these groups may require further studies. Also, in our cohort, there were only two patients with m-ACR A, which inherently limits the statistical significance achievable in categorizing this class separately. Nevertheless, differentiating the category A alone potentially has limited clinical relevance. Hence, we do not make any ACR A classification alone, but instead group them together with ACR B in the analyses. This is also clinically meaningful since ACR A and B typically do not have different risk profiles, prognoses, or patient management options. In the case of dense breast classification, 30% of patients (14 of 47 in m-ACR C) were inaccurately classified by g-SoS as non-dense breasts (A or B). Diagnosing ACR category C is indeed challenging because these breasts exhibit both dense and non-dense regions. This was also indicated by our inter-reader observations as well as in the literature [1]. While mammography and US are intended to examine the same anatomical part of the breast, any disparity between m-ACR and g-SoS in breast density classification may be attributed to potential shifts in analyzed regions of interest between these modalities, e.g., their projective vs. cross-sectional imaging natures as well as m-ACR assessing all breast quadrants while g-SoS in this study having been measured only in the UOQ. Furthermore, the differences between m-ACR and g-SoS in breast density classification may also arise from the subjective nature of mammography assessment. Accordingly, one could speculate the quantitative g-SoS measurements are comparatively more reliable and observer-independent, but this requires further studies to confirm with additional gold-standard measurement approaches such as MRI.

Our US-SoS technique utilizes a conventional transducer, covering only a cross-sectional slice of the breast, lacking comprehensive coverage. In our study, data was collected from two different probe orientations around the same location in the UOQ since fibroglandular tissue and mammary gland volumes and hence the quadrant percent breast density are known to be higher in the UOQ than in the other quadrants [35, 36], and the latest BI-RADS lexicon [1] instructs that the densest breast part should be used for breast density classification. Therefore, mammography-based breast density classification is mostly likely to depend on the fibroglandular tissue content in the UOQ. This is also clinically relevant because denser breast portions obscure the mammography sensitivity to inclusions [1], while such parts also present the highest risk for breast cancer [37, 38]. These earlier works are corroborated by our results showing that g-SoS measured in the UOQ alone correlates strongly with m-ACR assessed throughout the breast. This finding highlights the clinical potential of g-SoS with measurements at a single indicative location (UOQ). These results may also suggest that, even when dense fibroglandular tissue is observed locally in X-ray mammography, the breast SoS might be changing globally across the entire breast, including the UOQ that we observed. Further studies are required to test this and other hypotheses arising from our work, e.g., by increasing the number of measurement locations, such as different quadrants, for broader breast coverage. Combining g-SoS measurements from different quadrants could also further improve classification performance, which is already quite promising with measurements from UOQ alone in this work. Additionally, despite the operator placing the transducer on breast cross-sections without tumors (guided using B-Mode images), there is a possibility that an unnoticed tumor could lead to an artificial increase in g-SoS value. One should also note that m-ACR and g-SoS assess inherently different tissue properties, where the former assesses the visual tissue inhomogeneity, and the latter quantifies the physical value of sound speed.

In this study, we showed that g-SoS is a repeatable, quantitative, and non-ionizing biomarker, that is promising for breast density classification. Evaluating the breast density with g-SoS prior to mammography provides several advantages. The sensitivity of mammography decreases as breast density increases [1]. Therefore, especially for young women with an increased risk, SoS-based density assessment could help determine whether a mammogram is appropriate. If the patient has high breast density, it might then be preferred to include tomosynthesis in the diagnostics or to use US or MRI instead of mammography. Through repeated measurements over time, SoS-based density assessment could help to determine the best moment for a mammogram or MRI, particularly for women using an IUD, undergoing hormonal substitution, and/or without regular periods [39]. Additionally, breast density is an independent risk factor for breast cancer [3, 4]. SoS-based density assessment could be integrated into future risk calculation tools alongside other risk factors, to provide a more precise and personalized breast cancer risk assessment. This could help in primary care; for example, regular SoS-based breast density assessments during gynecological check-ups, combined with other risk factors, may provide improved cancer risk assessment or stratification for X-ray mammographic examination.

## Abbreviations

ACR: American College of Radiology
ANOVA: Analysis of variance
AUC: Area under the curve
BI-RADS: Breast Imaging Reporting and Data System
g-SoS: Global speed-of-sound
ICC: Intraclass correlation coefficient
IUD: Intrauterine device
m-ACR: Mammographic ACR
MAD: Mean absolute difference
MRI: Magnetic resonance imaging
ROC: Receiver operating characteristic
SoS: Speed-of-sound
UOQ: Upper-outer quadrant
US: Ultrasound

## References

[1] D’Orsi CJ, Sickles EA, Mendelson EB et al (2013) ACR BI-RADS^®^ Atlas, Breast Imaging Reporting and Data System. American College of Radiology, Reston, VA

[2] Kamangar F, Dores GM, Anderson WF (2006) Patterns of cancer incidence, mortality, and prevalence across five continents: defining priorities to reduce cancer disparities in different geographic regions of the world. J Clin Oncol 24:2137–2150 16682732 10.1200/JCO.2005.05.2308

[3] Brentnall AR, Harkness EF, Astley SM et al (2015) Mammographic density adds accuracy to both the Tyrer-Cuzick and Gail breast cancer risk models in a prospective UK screening cohort. Breast Cancer Res 17:1–10 26627479 10.1186/s13058-015-0653-5 PMC4665886

[4] Sak MA, Littrup PJ, Duric N, Mullooly M, Sherman ME, Gierach GL (2015) Current and future methods for measuring breast density: a brief comparative review. Breast Cancer Manag 4:209–221 28943893 10.2217/bmt.15.13 PMC5609705

[5] Harvey JA, Bovbjerg VE (2004) Quantitative assessment of mammographic breast density: relationship with breast cancer risk. Radiology 230:29–41 14617762 10.1148/radiol.2301020870

[6] Boyd NF, Martin LJ, Bronskill M, Yaffe MJ, Duric N, Minkin S (2010) Breast tissue composition and susceptibility to breast cancer. J Natl Cancer Inst 102:1224–1237 20616353 10.1093/jnci/djq239 PMC2923218

[7] McCormack VA, dos Santos Silva I (2006) Breast density and parenchymal patterns as markers of breast cancer risk: a meta-analysis. Cancer Epidemiol Biomarkers Prev 15:1159–1169 16775176 10.1158/1055-9965.EPI-06-0034

[8] Huo CW, Chew GL, Britt KL et al (2014) Mammographic density—a review on the current understanding of its association with breast cancer. Breast Cancer Res Treat 144:479–502 24615497 10.1007/s10549-014-2901-2

[9] Bae JM, Kim EH (2016) Breast density and risk of breast cancer in Asian women: a meta-analysis of observational studies. J Prev Med Public Health 49:367–375 27951629 10.3961/jpmph.16.054 PMC5160133

[10] Pace LE (2020) Dense breast notification legislation: more reasons for caution. J Gen Intern Med 35:1937–1939 32468433 10.1007/s11606-020-05708-2 PMC7351901

[11] U.S. Food and Drug Administration (2023) Mammography Quality Standards Act. U.S. Food and Drug Administration. Available via https://www.federalregister.gov/documents/2023/03/10/2023-04550/.mammography-quality-standards-act. Accessed 6 Nov 2024

[12] Pettersson A, Graff RE, Ursin G et al (2014) Mammographic density phenotypes and risk of breast cancer: a meta-analysis. J Natl Cancer Inst 106:1–11 10.1093/jnci/dju078 PMC4568991 24816206

[13] Boyd NF, Huszti E, Melnichouk O et al (2014) Mammographic features associated with interval breast cancers in screening programs. Breast Cancer Res 16:417 25346388 10.1186/s13058-014-0417-7 PMC4187338

[14] Melnikow J, Fenton JJ, Whitlock EP et al (2016) Supplemental screening for breast cancer in women with dense breasts: a systematic review for the U.S. preventive services task force. Ann Intern Med 164:268–278 26757021 10.7326/M15-1789 PMC5100826

[15] Bae MS, Moon WK, Chang JM et al (2014) Breast cancer detected with screening US: reasons for nondetection at mammography. Radiology 270:369–377 24471386 10.1148/radiol.13130724

[16] Boyd NF (2013) Mammographic density and risk of breast cancer. Am Soc Clin Oncol Educ Book 33:e57–e62 10.14694/EdBook_AM.2013.33.e57 23714456

[17] Berg WA, Blume JD, Cormack JB et al (2008) Combined screening with ultrasound and mammography vs mammography alone in women at elevated risk of breast cancer. JAMA 299:2151–2163 18477782 10.1001/jama.299.18.2151 PMC2718688

[18] Duric N, Littrup P, Poulo L et al (2007) Detection of breast cancer with ultrasound tomography: first results with the Computed Ultrasound Risk Evaluation (CURE) prototype. Med Phys 34:773–785 17388195 10.1118/1.2432161

[19] Ruiter NV, Zapf M, Hopp T et al (2012) 3D ultrasound computer tomography of the breast: a new era? Eur J Radiol 81:S133–S134 23083562 10.1016/S0720-048X(12)70055-4

[20] Duric N, Boyd N, Littrup P et al (2013) Breast density measurements with ultrasound tomography: a comparison with film and digital mammography. Med Phys 40:013501 23298122 10.1118/1.4772057 PMC3548830

[21] O’Flynn EA, Fromageau J, Ledger AE et al (2017) Ultrasound tomography evaluation of breast density: a comparison with noncontrast magnetic resonance imaging. Invest Radiol 52:343–348 28121639 10.1097/RLI.0000000000000347 PMC5417582

[22] Sak M, Duric N, Littrup P et al (2017) Using speed of sound imaging to characterize breast density. Ultrasound Med Biol 43:91–103 27692872 10.1016/j.ultrasmedbio.2016.08.021 PMC5761326

[23] Sanabria SJ, Goksel O, Martini K et al (2018) Breast-density assessment with handheld ultrasound: a novel biomarker to assess breast cancer risk and to tailor screening? Eur Radiol 28:3165–3175 29556766 10.1007/s00330-017-5287-9

[24] Ruby L, Sanabria SJ, Martini K et al (2019) Breast cancer assessment with pulse-echo speed of sound ultrasound from intrinsic tissue reflections: proof-of-concept. Invest Radiol 54:419–427 30913054 10.1097/RLI.0000000000000553

[25] Bezek CD, Goksel O (2023) Analytical estimation of beamforming speed-of-sound using transmission geometry. Ultrasonics 134:107069 37331051 10.1016/j.ultras.2023.107069

[26] Schweizer D, Rau R, Bezek CD, Kubik-Huch RA, Goksel O (2023) Robust imaging of speed-of-sound using virtual source transmission. IEEE Trans Ultrason Ferroelectr Freq Control 70:1308–1318 37549087 10.1109/TUFFC.2023.3303172

[27] Bland JM, Altman DG (1986) Statistical methods for assessing agreement between two methods of clinical measurement. Lancet 327:307–310 2868172

[28] Koo TK, Li MY (2016) A guideline of selecting and reporting intraclass correlation coefficients for reliability research. J Chiropr Med 15:155–163 27330520 10.1016/j.jcm.2016.02.012 PMC4913118

[29] Overholser BR, Sowinski KM (2008) Biostatistics primer: part 2. Nutr Clin Pract 23:76–84 18203967 10.1177/011542650802300176

[30] Checka CM, Chun JE, Schnabel FR, Lee J, Toth H (2012) The relationship of mammographic density and age: implications for breast cancer screening. AJR Am J Roentgenol 198:W292–W295 22358028 10.2214/AJR.10.6049

[31] Glide C, Duric N, Littrup P (2007) Novel approach to evaluating breast density utilizing ultrasound tomography. Med Phys 34:744–753 17388192 10.1118/1.2428408

[32] Irshad A, Leddy R, Ackerman S et al (2016) Effects of changes in BI-RADS Density Assessment Guidelines (fourth versus fifth edition) on breast density assessment: intra- and interreader agreements and density distribution. AJR Am J Roentgenol 207:1366–1371 27656766 10.2214/AJR.16.16561

[33] Bamber JC, Hill CR (1979) Ultrasonic attenuation and propagation speed in mammalian tissues as a function of temperature. Ultrasound Med Biol 5:149–157 505616 10.1016/0301-5629(79)90083-8

[34] Weiwad W, Heinig A, Goetz L et al (2000) Direct measurement of sound velocity in various specimens of breast tissue. Invest Radiol 35:721–726 11204798 10.1097/00004424-200012000-00005

[35] Shim S, Unkelbach J, Landsmann A, Boss A (2023) Quantitative study on the breast density and the volume of the mammary gland according to the patient’s age and breast quadrant. Diagnostic (Basel) 13:3343 10.3390/diagnostics13213343 PMC10648521 37958239

[36] Chen JH, Liao F, Zhang Y et al (2017) 3D MRI for quantitative analysis of quadrant percent breast density: correlation with quadrant location of breast cancer. Acad Radiol 24:811–817 28131498 10.1016/j.acra.2016.12.016 PMC5482764

[37] Ursin G, Hovanessian-Larsen L, Parisky YR, Pike MC, Wu AH (2005) Greatly increased occurrence of breast cancers in areas of mammographically dense tissue. Breast Cancer Res 7:R605–R608 16168104 10.1186/bcr1260 PMC1242126

[38] Pinto Pereira SM, McCormack VA, Hipwell JH et al (2011) Localized fibroglandular tissue as a predictor of future tumour location within the breast. Cancer Epidemiol Biomarkers Prev 20:1718–1725 21693627 10.1158/1055-9965.EPI-11-0423 PMC3154655

[39] Ruby L, Sanabria SJ, Obrist AS et al (2019) Breast density assessment in young women with ultrasound based on speed of sound: influence of the menstrual cycle. Medicine (Baltimore) 98:e16123 31232962 10.1097/MD.0000000000016123 PMC6636937

